# Multifaceted Biomedical Applications of Functional Graphene Nanomaterials to Coated Substrates, Patterned Arrays and Hybrid Scaffolds

**DOI:** 10.3390/nano7110369

**Published:** 2017-11-04

**Authors:** Yong Cheol Shin, Su-Jin Song, Suck Won Hong, Seung Jo Jeong, Wojciech Chrzanowski, Jae-Chang Lee, Dong-Wook Han

**Affiliations:** 1 Research Center for Energy Convergence Technology, Pusan National University, Busan 46241, Korea; choel15@naver.com; 2 Department of Cogno-Mechatronics Engineering, College of Nanoscience & Nanotechnology, Pusan National University, Busan 46241, Korea; songsj86@gmail.com (S.-J.S.); swhong@pusan.ac.kr (S.W.H.); 3 GS Medical Co., Ltd., Cheongju-si, Chungcheongbuk-do 28161, Korea; eric.jeong@gsmedi.com; 4 Australian Institute for Nanoscale Science and Technology, Charles Perkins Centre, Faculty of Pharmacy, University of Sydney, Pharmacy and Bank Building A15, Sydney NSW 2006, Australia; wojciech.chrzanowski@sydney.edu.au; 5 Research Center for Industrial Chemical Biotechnology, Korea Research Institute of Chemical Technology, Ulsan 44429, Korea

**Keywords:** graphene nanomaterial, multifaceted biomedical application, coated substrate, patterned array, hybrid scaffold

## Abstract

Because of recent research advances in nanoscience and nanotechnology, there has been a growing interest in functional nanomaterials for biomedical applications, such as tissue engineering scaffolds, biosensors, bioimaging agents and drug delivery carriers. Among a great number of promising candidates, graphene and its derivatives—including graphene oxide and reduced graphene oxide—have particularly attracted plenty of attention from researchers as novel nanobiomaterials. Graphene and its derivatives, two-dimensional nanomaterials, have been found to have outstanding biocompatibility and biofunctionality as well as exceptional mechanical strength, electrical conductivity and thermal stability. Therefore, tremendous studies have been devoted to employ functional graphene nanomaterials in biomedical applications. Herein, we focus on the biological potentials of functional graphene nanomaterials and summarize some of major literature concerning the multifaceted biomedical applications of functional graphene nanomaterials to coated substrates, patterned arrays and hybrid scaffolds that have been reported in recent years.

## 1. Introduction

Over the last several decades, the development of nanoscience and nanotechnology has made dramatic progress in research towards the understanding of nanomaterials. In addition, numerous attempts have been devoted at that time to employ nanomaterials in various research and industrial fields. It is commonly acknowledged that nanomaterials are defined as materials having at least one dimension, such as length, thickness, width, or diameter, of smaller than 100 nm in size and the plenty of attentions has been paid to various nanomaterials, including carbon nanotube, nanoparticle, polymeric nanofiber, quantum dot, graphene and nanocomposite [1,2,3,4]. Nanomaterials exhibit unique properties, including extraordinary physicochemical, fluorescent, electrical and thermomechanical properties that do not appear in those bulk counterparts of the same composition [5,6,7,8,9,10,11]. Moreover, a very large surface area-to-volume ratio is probably the most distinctive property of nanomaterials, which makes them even more attractive for applications in diverse fields, such as physics, chemistry, biology, material science and electrical engineering. Therefore, many researchers have been interested in the unlimited potentials of nanomaterials and extensively studying to apply them to each research field.

Among a great number of nanomaterials, graphene is a relatively recently discovered and characterized nanomaterial that has a variety of advantages and has particularly attracted renewed interest in nanomaterial research. Graphene, a single atomic layer of graphite, is a two-dimensional (2D) nanomaterial, in which sp^2^-bonded carbon atoms arranged in a honeycombed lattice structure. It was first described by Boehm and coworkers in 1986 [12]. After that, it was identified and isolated by Geim and Novoselov in 2004 and has been receiving an explosion in attention [13]. Graphene is a novel nanomaterial with remarkable properties, such as exceptional thermomechanical, excellent physicochemical, outstanding electrical and unique biological properties and its applicable fields are endless [14,15,16,17,18,19]. In addition to graphene, graphene derivatives—including graphene oxide (GO) and reduced graphene oxide (rGO)—also possess promising potentials as a graphene because they have many functional groups on their surface while maintaining the unique properties of graphene. GO can be obtained by oxidizing graphene and has many oxygen-containing functional groups, such as hydroxyl, carboxyl, epoxy and carbonyl groups, on its surface, which leads to facilitative interactions with biomolecules or cells. On the other hand, rGO can be prepared by reducing GO. rGO has been also actively studied because it has oxygen-containing functional groups on its surfaces as well as having relatively superior electrical properties as compared with GO [20,21,22]. Due to the obvious advantages of graphene and graphene derivatives, numerous studies on their toxic and biological effects have been reported and their applications to the biomedical fields have been constantly explored [17,23,24,25,26,27]. According to the recent findings, graphene and its derivatives have been revealed to have not only outstanding biocompatibility but also ability to enhance cellular behaviors, including cell growth, proliferation and differentiation [28,29,30,31,32,33,34,35,36]. However, unfortunately, there has been a lot of debate about the biosafety and biological effects of graphene and graphene derivatives, although much research has been conducted on these issues. In particular, the interactions of graphene and graphene derivatives with biological systems are quite varied depending on many parameters, including their size, shape, concentration, surface functional group, exposure time and preparation method; thereby, research on the biomedical applications of graphene nanomaterials is still in its infancy.

Herein, we focus on the key literature concerning the biomedical applications of graphene and its derivatives and are attempting to present valuable information that can provide guidance for future comprehensive research on their biomedical applications. Tremendous studies have been underway to employ graphene and graphene derivatives for biomedical applications by introducing functional groups on their surface, or by using them as surface coating, nanofiller and composite materials. Among these various types of applications, the aim of the present review is to summarize the recent studies concerning multifaceted biomedical applications of functional graphene nanomaterials to coated substrates, patterned arrays and hybrid scaffolds.

## 2. Graphene Nanomaterial-Coated Substrates

**Figure 1:**
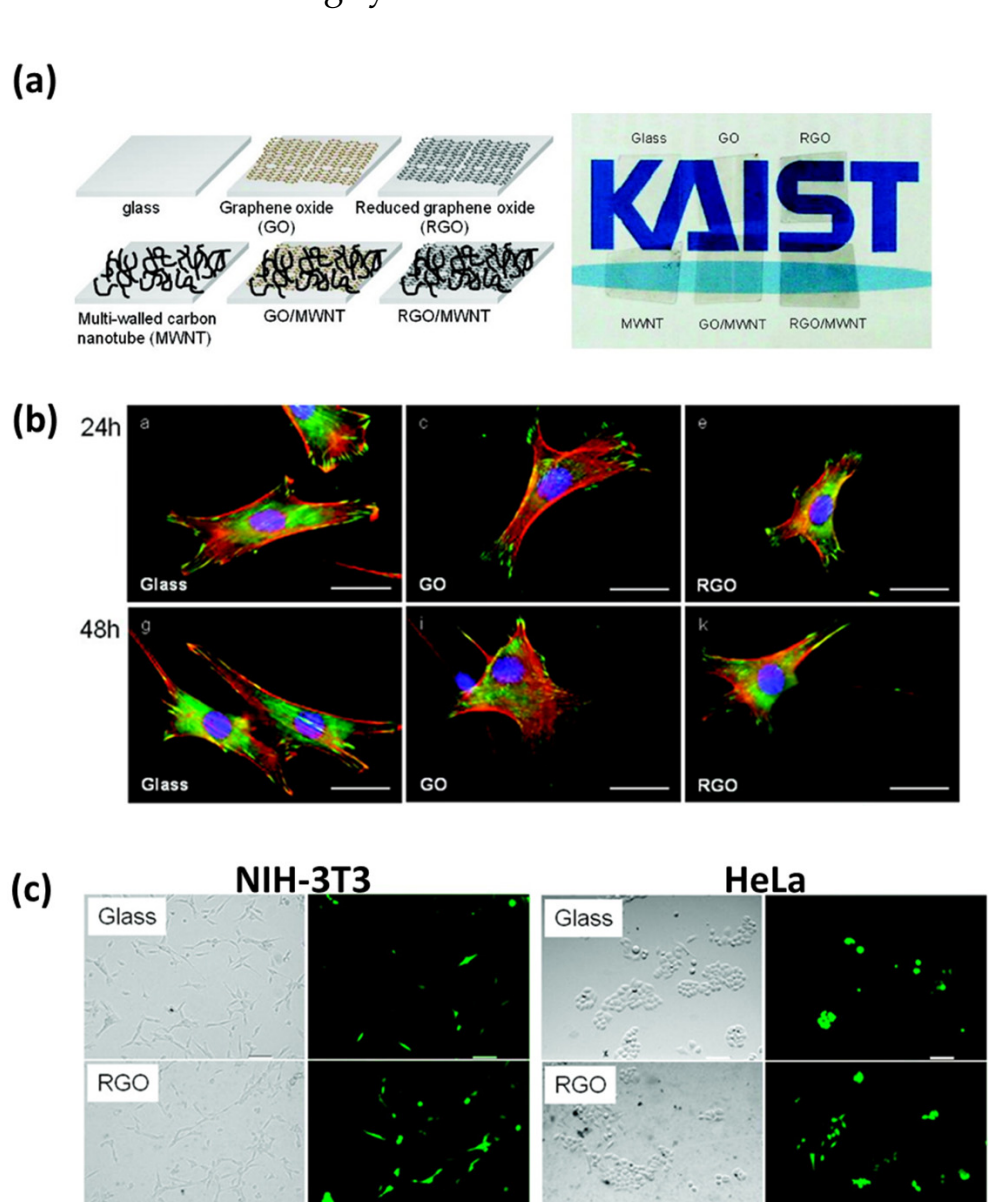
Stimulating effects of graphene nanomaterial-coated substrates on NIH-3T3 fibroblast behaviors. (**a**) Structural diagrams (left panel) and optical images (right panel) of graphene nanomaterial-coated substrate. (**b**) The fluorescence images of NIH-3T3 fibroblasts on each substrate for 24 and 48 h. Scale bars are 20 μm. (**c**) Improved gene transfection efficiency of NIH-3T3 fibroblasts and HeLa cells on rGO-coated substrates after 48 h incubation. Scale bars are 35 μm. Reproduced with permission from [44]. American Chemical Society, 2010.

**Figure 2:**
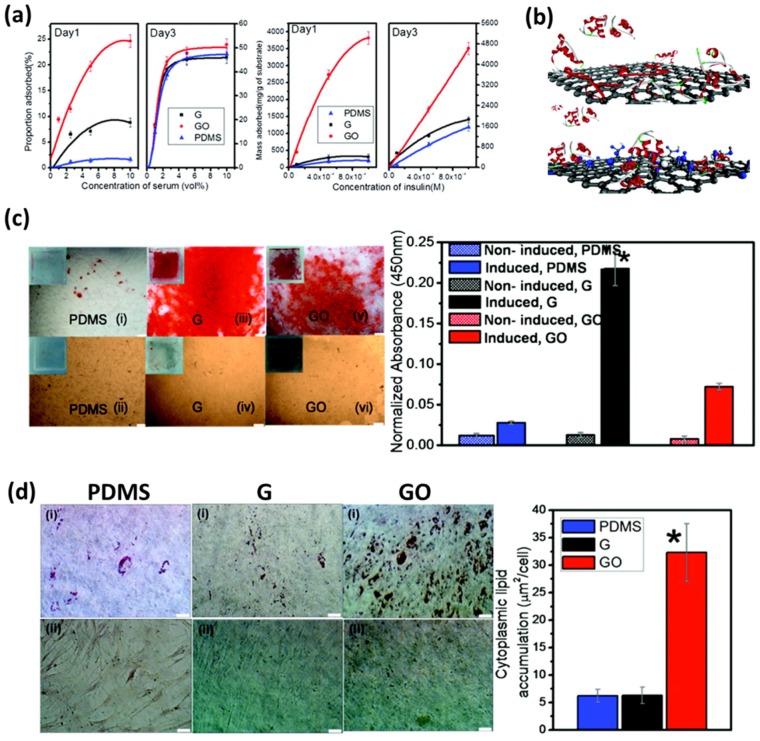
Molecular origin of enhanced cell behaviors on graphene nanomaterial-coated substrates. (**a**,**b**) Different binding interactions of graphene and GO with serum and insulin. (**c**) Osteogenic differentiation of MSCs after 12 days of incubation on PDMS (i) with induction and (ii) without induction, on graphene (iii) with induction and (iv) without induction and on GO (v) with induction (vi) and without induction. Scale bars are 200 μm. (**d**) Adipogenic differentiation of MSCs after 14 days of induction on PDMS (i) with induction and (ii) without induction, on graphene (i) with induction and (ii) without induction and on GO (i) with induction and (ii) without induction. Scale bars are 50 μm. Reproduced with permission from [29]. Copyright American Chemical Society, 2011.

Until now, it has been widely believed that the toxicity of graphene nanomaterials is quite different depending on their shape, size, concentration, surface functional group and preparation method [37,38,39]. In particular, the biological effects of graphene nanomaterials have been reported to be strongly dependent on size, concentration, exposure time and cell type [37,38,39,40,41,42,43]. Therefore, many studies have been suggested to improve cell behaviors by coating graphene nanomaterials on substrates to minimize the influence of those various parameters (Figure 1 and Figure 2) [28,29,30,32,44,45,46,47,48,49,50,51,52]. Ryoo et al. reported that the graphene nanomaterials, including GO and rGO, can be simply immobilized onto glass substrates treated with 3-aminopropyltriethoxysilane (3-APTES) via electrostatic interactions between GO and amine groups on the substrates (Figure 1a) [44]. In addition, they investigated the behaviors of NIH-3T3 fibroblasts on the graphene nanomaterial-coated substrates and revealed that the GO- and rGO-coated glass substrates can not only favorably support cell adhesion, spreading and proliferation, but can also improve the gene transfection efficiency of cells as compared to the glass substrates without graphene nanomaterial coatings (Figure 1b,c). These results indicated that the graphene nanomaterial-coated substrates are highly cell-friendliness and the graphene nanomaterials can be readily employed as surface coating materials in biomedical applications, such as implant, cell culture platform and cell-interfacing system.

Moreover, these stimulating effects of graphene nanomaterial-coated substrates on cell behaviors have also been examined in various cell types, including mesenchymal stem cells (MSCs), neural stem cells (NSCs), induced pluripotent stem cells (iPSCs) and myoblasts. Nayak et al. documented that the graphene coatings on substrates can effectively accelerate the differentiation of human MSCs without hampering cell proliferation. The human MSCs were successfully differentiated into osteogenic lineages only on graphene-coated regions. Moreover, it was shown that the graphene-coated polymeric substrates, including polydimethylsiloxane (PDMS) and polyethylene terephthalate (PET), could also increase osteogenic differentiation. In general, cellular behaviors are strongly dependent on the stiffness of substrates and the osteogenic differentiation is commonly promoted on stiff substrates rather than soft substrates, such as polymeric substrates [53,54,55,56]. However, the differentiation of human MSCs towards osteogenic lineage was increased on graphene-coated polymeric substrates (i.e., soft substrates) regardless of stiffness of underlying substrates, indicating that the graphene coatings can be a driving force of osteogenic differentiation.

Meanwhile, Lee et al. investigated the molecular origin of accelerated differentiation on graphene nanomaterial-coated substrates by comparing the binding abilities of graphene and GO to different growth factors [29]. They showed that the different binding interactions of graphene and GO with growth factor agents play a significant role in determining the stem cell growth and differentiation (Figure 2a,b). The osteogenic differentiation of human bone marrow-derived MSCs was enhanced on the graphene-coated PDMS substrates through π-π stacking interactions between graphene and osteogenic inducer, including dexamethasone and β-glycerophosphate, while GO-coated PDMS substrates could greatly enhance adipogenic differentiation via hydrogen bonding and electrostatic interactions with insulins (Figure 2c,d).

**Figure 3:**
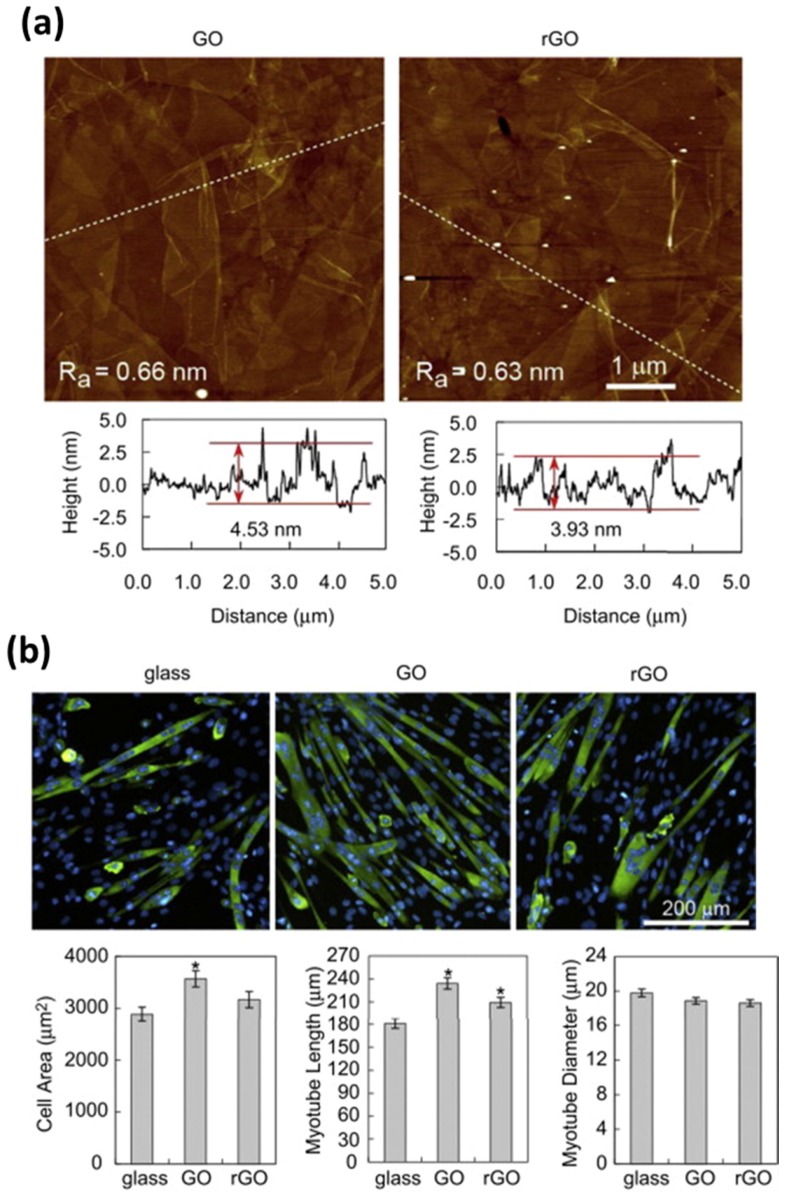
Enhanced growth and differentiation of myoblasts on graphene nanomaterial-coated substrates. (**a**) Characterizations of GO- and rGO-coated glass substrates by atomic force microscopy (AFM). (**b**) Myogenic differentiation on uncoated, GO- and rGO-coated glass substrates. C2C12 cells were grown in growth media (Dulbecco’s modified Eagle’s medium, DMEM, containing 10% fetal bovine serum and 1% antibiotic-antimyotic solution) for 1 day and then incubated in differentiation media (DMEM containing 2% horse serum and 1% antibiotic-antimyotic solution) for 5 days. Reproduced with permission from [32]. Copyright Elsevier Ltd, 2012.

The specific binding affinity of GO for biomolecules can significantly promote the myoblast growth and myogenic differentiation. Ku et al. studied the myoblast behaviors on GO- and rGO-coated glass substrates and indicated that the GO- and rGO-coated substrates could enhance myogenic differentiation as well as supporting cell adhesion and proliferation (Figure 3) [32]. They suggested that the enhanced myogenic differentiation was attributed to both the unique physicochemical properties of graphene derivatives, such as ripples and wrinkles and the adsorption ability for serum proteins in culture media. Moreover, it was confirmed that the GO-coated substrates were more favorable for myogenic differentiation because GO has more oxygen-containing functional groups on its surface than rGO, which leads to further increase in serum protein adsorption.

In other studies, the graphene nanomaterial-coated substrates hold the potentials for neural cells [30,46,48,57]. In particular, it is worth noting that the graphene nanomaterials have superior electrical properties as compared with the standard graphite materials [58,59]. Qiu et al. demonstrated that the conductivity of graphene nanomaterials is quite a bit higher than that of graphite materials (highly oriented pyrolytic graphite crystal, HOPG), while the resistivity of graphene nanomaterials is much lower as compared with that of graphite materials [59]. They described that the conductivities were found to be 92, 407 and 2138 (Ω·cm)^−1^ for the HOPG sample, the multi-layer graphene sample and the sample with a single-layer to few-layer graphene, respectively. On the other hand, there is also a significant increase in the carrier concentration, especially in the samples containing a mixture of single-layer to several layers of graphene: 3.58 × 10^18^, 14.9 × 10^18^ and 46.3 × 10^18^ cm^−3^ for HOPG sample, multi-layer graphene sample and samples containing a mixture of single-layer to several layers of graphene, respectively. In addition, it was revealed that the superior electrical properties of graphene nanomaterials result in highly sensitive detection of the micromolar concentration of dopamine—a neurotransmitter—on graphene-coated surfaces by Raman spectroscopy and microscopy.

**Figure 4:**
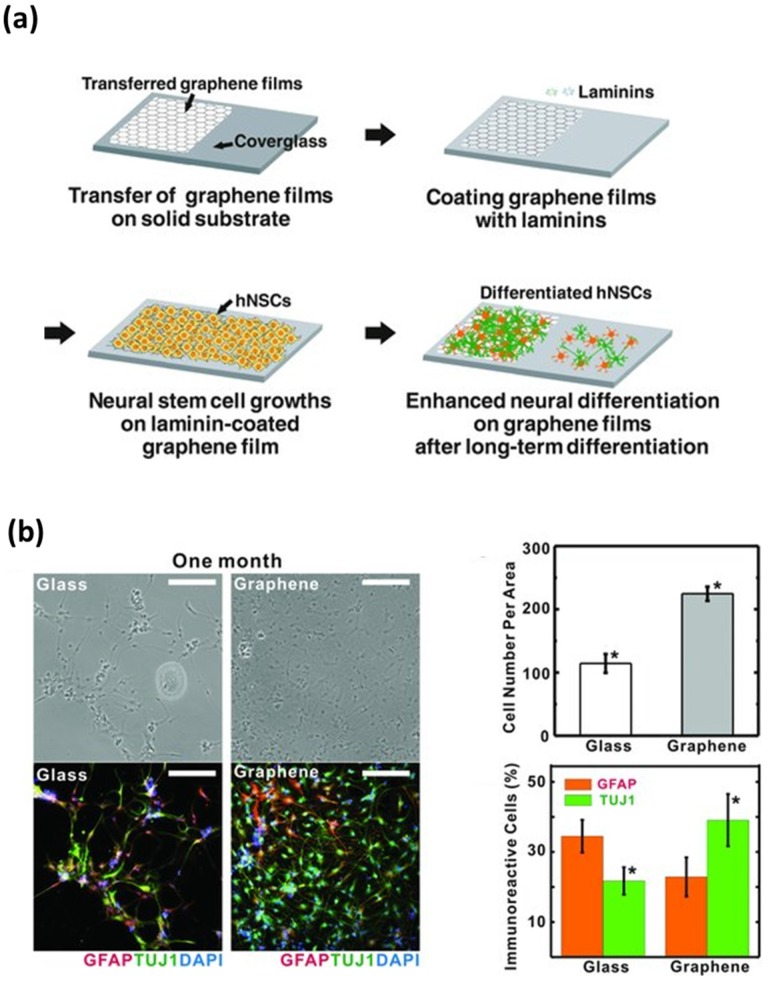
Enhanced growth and differentiation of human NSCs on graphene nanomaterial-coated substrates. (**a**) Schematic diagram depicting the growth and differentiation of human NSCs on graphene. (**b**) Enhanced neural differentiation of human NSCs on graphene-coated substrates. Scale bars are 200 μm. Reproduced with permission from [30]. Copyright John Wiley and Sons, 2011.

Moreover, these superior electrical properties also allow graphene nanomaterials to be used in stimulating neural cells. Park et al. found that the differentiation of human NSCs into neurons was increased on graphene-coated substrates (Figure 4). Meanwhile, Tang et al. cultured NSCs on graphene-coated substrates and investigated the neural excitation by monitoring spontaneous Ca^2+^ oscillations, which represents neural signal transmission [57]. The results indicated that the NSCs were able to form functionally active neural networks on graphene-coated substrates and the neural network activities, such as the intracellular spontaneous and synchronous Ca^2+^ oscillations and spontaneous synaptic currents, were significantly improved on the graphene-coated substrates. Hence, it is indicated that graphene nanomaterial-coated substrates are a typical strategy for biomedical applications of graphene nanomaterials.

In addition to these, graphene-coated substrates or materials can be also applied for other biomedical applications, such as biosensor, implantable electrode, antibacterial system and composite graft material [50,51,52,60,61,62,63,64,65]. Several studies related to the other biomedical applications of graphene nanomaterial-coated substrates are summarized in Table 1.

## 3. Graphene Nanomaterial-Patterned Arrays

Up to now, much research has indicated that the beneficial effects of graphene nanomaterials in biomedical applications, such as promoting effects on cellular behaviors, including cell adhesion, proliferation, development, spreading and differentiation. Along with those findings previously reported, there have been considerable efforts to use the unique physicochemical and topographical properties of graphene nanomaterials in biomedical applications [64,66,67]. In particular, distinctive rippled or wrinkled features of graphene nanomaterials can provide specific topographical guidance cues for directing cell behaviors. The precisely controlled cell migration or orientation plays a crucial role in determining cell responses and fates [68,69,70]. Therefore, the studies concerning the regulation of cellular behaviors by graphene nanomaterials have been recently proposed and investigated for biomedical applications.

**Figure 5:**
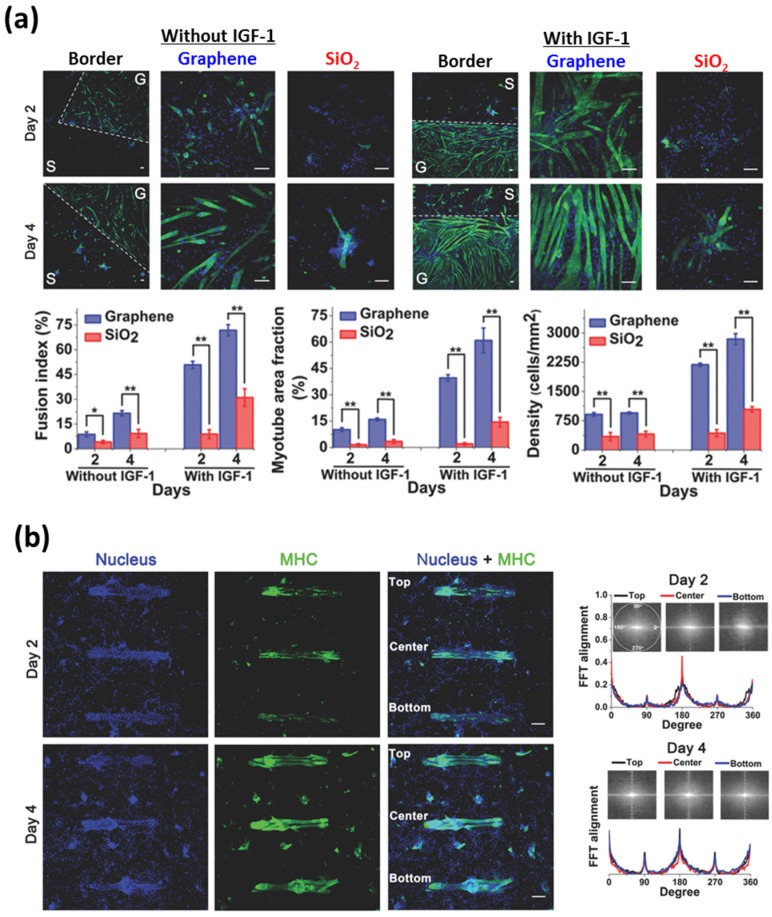
Graphene-based patterning and differentiation of myoblasts. (**a**) Myogenic differentiation of C2C12 skeletal muscle myoblasts on graphene-patterned substrates. The dashed white line in column one indicates the border of SiO_2_ and graphene surfaces on the substrates. Scale bars are 100 μm. (**b**) Fluorescence images and alignment of the C2C12 myotubes on rectangular island-shaped graphene patterns. Scale bars are 250 μm. Reproduced with permission from [71]. Copyright John Wiley and Sons, 2013.

The graphene nanomaterial-patterned arrays have been especially spotlighted as a novel strategy for guiding and stimulating cellular behaviors, because the graphene nanomaterials can provide desirable topographical guidance cues as well as biochemical cues [71,72,73,74,75,76,77,78,79]. Bajaj et al. fabricated rectangular island-shaped graphene patterns on SiO_2_/Si substrate using photolithography techniques and examined the myogenic differentiation of C2C12 skeletal muscle myoblasts (Figure 5) [71]. It was shown that most myotubes were formed on graphene patterns, while few cells were differentiated into myotubes on the SiO_2_/Si substrate without graphene patterns (Figure 5a). In addition, the island-shaped graphene patterns were able to induce the spontaneous alignment of myotubes, which leads to a maximized the contractile power for muscle contractions (Figure 5b). Moreover, they evaluate the functionality of myotubes on graphene patterns and revealed that the myotubes on graphene patterns were mature and highly functional.

**Figure 6:**
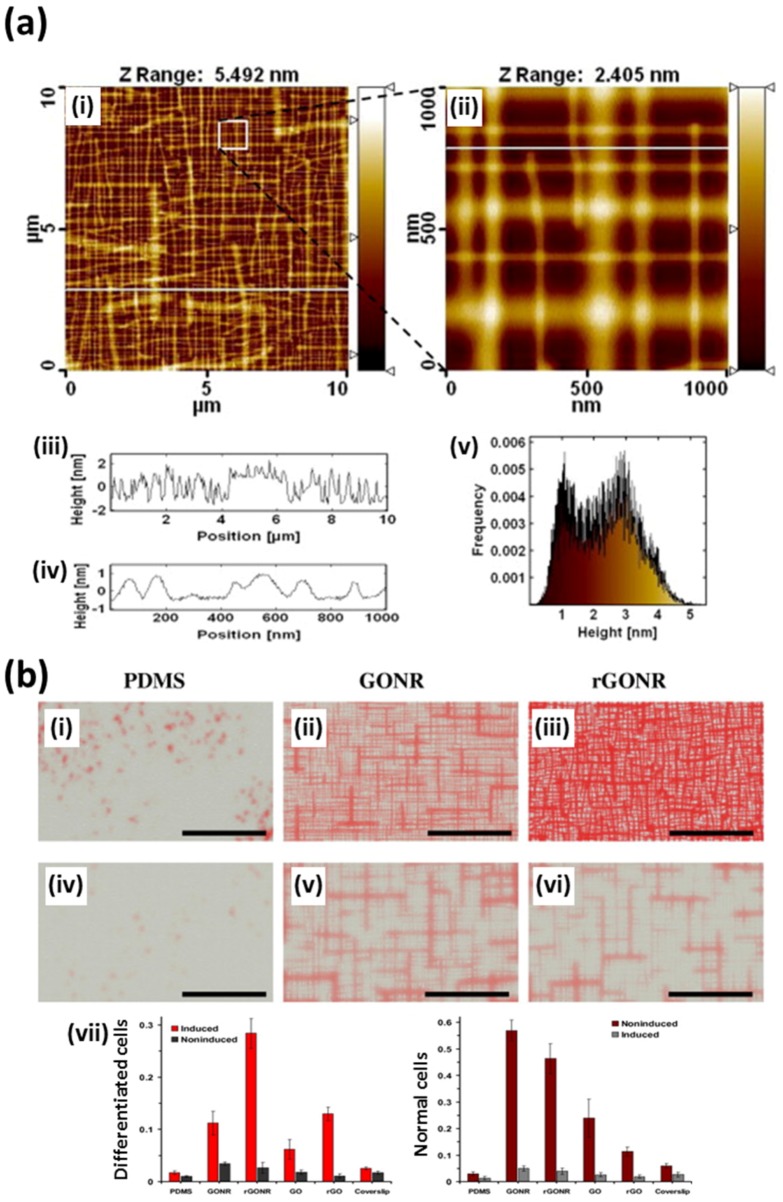
Graphene-based patterning and differentiation of human MSCs. (**a**) Characterizations of aligned GO nanoribbon grid on Si_3_N_4_/Si(100) substrates by AFM. AFM images of GO nanoribbon grid in a (i) wide and (ii) close window. (iii) and (iv) present height profiles of GO nanoribbon grid along the white lines marked in (i) and (ii), respectively. (v) exhibits the height profile histogram of GO nanoribbon grid. (**b**) Accelerated osteogenic differentiation of human MSCs determined by Alizarin Red staining after 1 week incubation (i–iii) with induction and (iv–vi) without induction. Scale bars are 10 μm. (vii) Normalized optical absorbance of the differentiated cells and normal cells. Reproduced with permission from [72]. Copyright Elsevier Ltd, 2013. Abbreviations: G, graphene; GONR, GO nanoribbon grid; MHC, myosin heavy chain; rGONR, rGO nanoribbon grid; S, SiO_2_.

Akhavan et al. have also demonstrated that graphene patterns can be employed as selective 2D templates for accelerating the osteogenic differentiation of human MSCs (Figure 6) [72]. They fabricated aligned GO and rGO nanoribbon grid on Si_3_N_4_/Si(100) substrates by a paint-brushing method and investigated the osteogenic differentiation of human MSCs (Figure 6a). Their results indicated that both graphene nanogrids (GO and rGO nanoribbon grid) could enhance the actin cytoskeleton proliferations. Meanwhile, in the presence of chemical inducers, including dexamethasone, β-glycerophosphate and ascorbic acid, rGO nanoribbon grid especially accelerated the osteogenic differentiation of human MSCs (Figure 6b). They explained these findings by the fact that the rGO nanoribbon grids can highly adsorb chemical induces in culture media and can also provide physical stress induced by the surface topographical features of rGO nanogrids [29,80,81,82]. They also proved that those stimulating effects of rGO nanogrids are equally effective on human NSCs [73]. These results indicate that the graphene nanomaterial patterns can be readily applied in biomedical fields.

**Figure 7:**
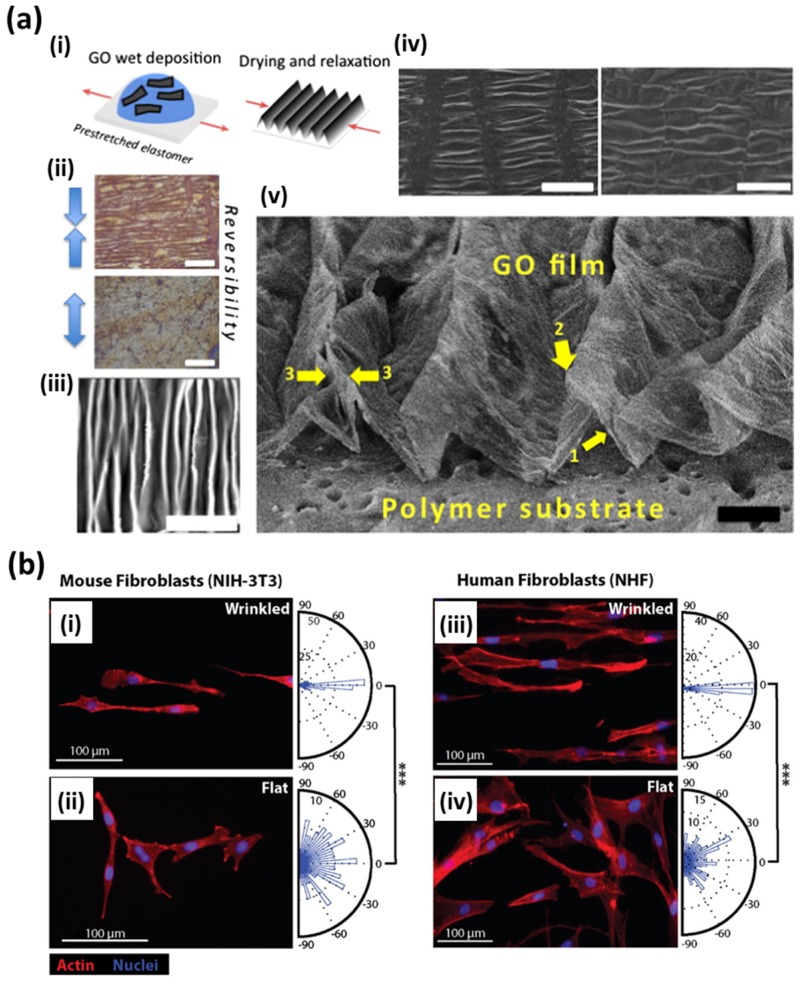
Controlling cellular behaviors using graphene nanomaterial-patterned arrays. (**a**) Fabrication process and morphology of wrinkled GO multilayer films. (i) Schematic diagram and (ii) reversibility of the fabrication process. Scale bars are 100 μm. (iii) AFM image, (iv) scanning electron microscopy (SEM) images and (v) high-resolution SEM image of wrinkled GO patterns. Scale bar are 20, 100 and 20 μm for (iii), (iv) and (v), respectively. (**b**) Fluorescence images of MIH-3T3 cells and human fibroblasts on (i,iii) 200 nm wrinkled GO substrates and (ii,iv) flat graphene substrates. Reproduced with permission from [77]. Copyright Elsevier Ltd, 2015.

In several studies described above, the excellent biocompatibility and the applicability of graphene nanomaterial patterns in biomedical applications have been demonstrated. However, such patterning of graphene nanomaterials requires elaborate techniques, such as photolithography, dip-pen lithography and microcontact printing. These techniques, of course, are sufficiently efficient but simpler and more scalable methods have been reported by Wang et al. (Figure 7a) [77]. They simply fabricated wrinkled GO multilayer films by relaxation of GO sheets on pre-stretched elastomers. The wrinkled GO patterns can be easily removed by re-stretching the elastomer substrates and the fabrication process is reversible. In addition, the wavelength and height of GO wrinkled can be controlled by film thickness and pre-stretch. The cell alignment and morphology on the wrinkled GO patterns were also evaluated. It was observed that the fabricated GO wrinkles can effectively induce cell alignment and elongation by contact guidance provided from wrinkled GO patterns (Figure 7b). Hence, it was suggested that the wrinkled GO patterns are promising new approach for functional biomedical applications due to advantages, such as the simplicity and scalability of fabrication.

**Figure 8:**
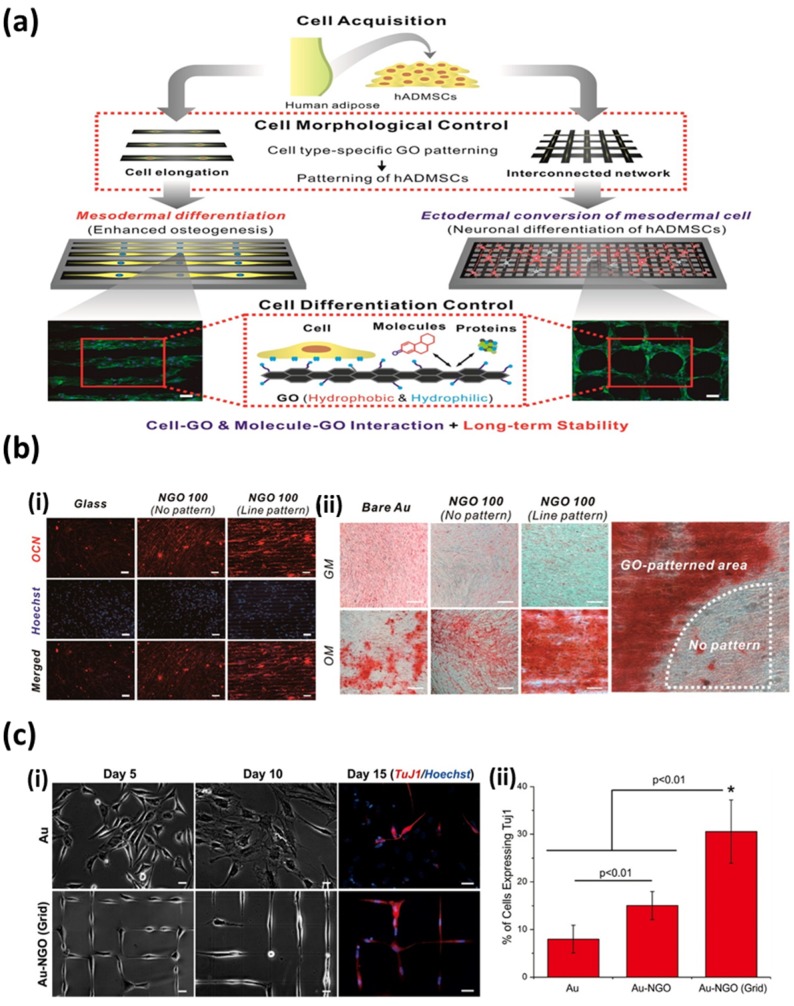
**(a)** Schematic diagram depicting control over the differentiation of human ADMSCs using the geometries of GO-patterned arrays. (**b**) Enhanced osteogenic differentiation of human ADMSCs using GO line-patterned arrays. (i) Fluorescence images of human ADMSCs on GO line-patterned arrays, showing elongated and well-spread morphology of human ADMSCs. Scale bars are 50 μm. (ii) Enhanced osteogenic differentiation of human ADMSCs confirmed by Alizarin Red staining at day 21. Scale bars are 50 μm. (**c**) Encouraged neuronal differentiation of human ADMSCs using GO grid-patterned arrays. (i) Fluorescence images of neural induced human ADMSCs on poly-L-lysine (PLL)-coated Au and GO grid-patterned arrays. Scale bars are 20 μm. (ii) Quantitative analysis of the percentage of cell expressing the neuronal marker (TuJ1). Reproduced with permission from [76]. Copyright American Chemical Society, 2015.

On the other hand, intriguing results have been obtained by Kim et al. [76]. Kim et al. ascertained that the stem cell fate can be controlled by manipulating the sizes and geometries of patterned arrays (Figure 8a). They prepared GO-patterned arrays with different sizes and geometries on various types of substrates and examined the differentiation of human adipose-derived mesenchymal stem cells (ADMSCs) on those GO-patterned arrays. Interestingly, the differentiation of human ADMSCs was observed to strongly depend on the geometries of GO-patterned arrays. The GO line patterns promote the elongation and spreading of human ADMSCs following the geometry of GO line patterns, which results in the enhanced osteogenesis of human ADMSCs (Figure 8b). On the contrary, the GO grid patterns guide human ADMSCs to grow in a bipolar orientation and encourage the conversion of mesodermal stem cells to ectodermal neuronal cells (Figure 8c). These different cellular behaviors of human ADMSCs could be attributed to the physicochemical and geometric properties of GO-patterned arrays, indicating that the graphene nanomaterial-patterned arrays are particularly attractive for biomedical applications.

These guidance effects of graphene nanomaterial-patterned arrays are closely related to their size and shape. Zhang et al. confirmed that the width of GO-patterned arrays can directly affect the cell migration, alignment, morphology and cell adhesion [78]. They found that the cytoskeleton contractility, intracellular traction and actin filament elongation are significantly enhanced when the width of the GO-patterned arrays is similar to the cell dimension, which in turn cell migration is greatly increased. Kim et al. also revealed that the shape of GO-patterned arrays can determine cell morphology, migration distance, speed and directionality [79]. Therefore, graphene nanomaterial-patterned arrays fabricated with sophisticated control of structures and properties can provide unique opportunities for biomedical applications.

## 4. Graphene Nanomaterial-Based Hybrid Scaffolds

There have been astonishing advances in biomedical applications of graphene nanomaterials but at the same time, still there are many challenges remain to be solved. To employ graphene nanomaterials in biomedical applications, ensuring biosafety and biocompatibility of graphene nanomaterials is the first priority. To address this issue, there have been substantial efforts to develop graphene nanomaterials-based scaffolds through hybridization with biocompatible materials to maintain the unique properties of graphene nanomaterials and to improve biocompatibility [31,83,84,85,86,87,88,89,90,91,92,93,94,95,96,97,98,99,100,101].

**Figure 9:**
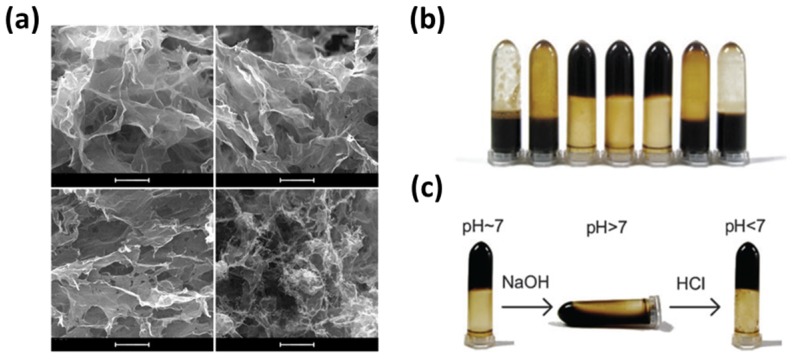
pH-sensitive GO/PVA composite hydrogel. (**a**) SEM images of GO/PVA composite hydrogel. Scale bars are 5 μm. (**b**) Digital photographs of GO/PVA composite hydrogel with different content ratio (*r*_P/G_). From left to right, *r*_P/G_ = 1:1, 1:1.5, 1:2, 1:5, 1:10, 1:20 and 1:40. (**c**) pH-sensitive gel-sol-gel transition behaviors according to the pH values. From left to right, pH ≈ 7, pH > 7 and pH < 7, respectively. Reproduced with permission from [83]. Copyright The Royal Society of Chemistry, 2010.

Bai et al. reported that the GO and poly(vinyl alcohol) (PVA) composite hydrogels can be easily prepared and the GO/PVA composite hydrogels showed pH-sensitive gel-sol-gel transition behaviors (Figure 9) [83]. The GO/PVA composite hydrogels were decomposed with increasing pH value and gel-sol transition occurred. Meanwhile, when the pH value dropped again, the GO/PVA composite hydrogels underwent sol-gel transition. This could be attributed to the surface negative charge of GO originated from the carboxyl groups. The different pH values led to changes in surface charge densities of GO, which in turn, electrostatic repulsion forces between GO sheets were altered. These pH-sensitive properties make the GO/PVA composite hydrogels exceptionally useful as drug delivery carriers. On the other hand, graphene nanomaterial-hybridized hydrogels can be also utilized as cell scaffolds. Cha et al. showed that the mechanical properties of methacrylated gelatin (GelMA) hydrogels can be controlled by incorporation of methacrylate group-introduced GO (MeGO) and the GO-incorporated GelMA hydrogels showed good biocompatibility with fibroblasts [86]. The fracture strength of GelMA hydrogels could be enhanced by incorporating MeGO, while minimizing the changes in their rigidity. These enhanced mechanical properties were due to the interfacial bonding between GO and polymeric network [102,103]. In addition, the incorporated GO or MeGO did not detrimental effects on the viability and proliferation of encapsulated fibroblasts. Qiu et al. also proved the improving effects of graphene incorporation on the mechanical performance of polymeric hydrogels. They introduced the graphene aerogel into the poly(*N*-isopropylacrylamide) (PNIPAM) hydrogels. The results demonstrated that the good mechanical strength and electrical conductivity of graphene can significantly increase the mechanical performance of polymer hydrogels, suggesting that graphene nanomaterials can be used as reinforcing nanofillers for polymeric composites.

**Figure 10:**
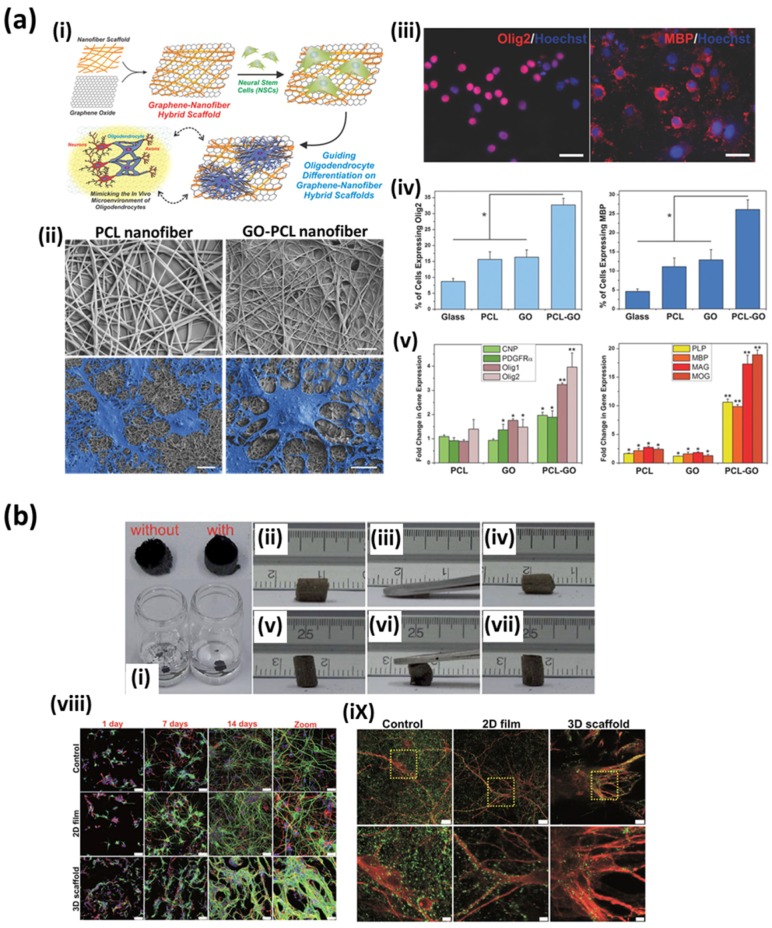
Multifaceted biomedical applications of functional graphene nanomaterials to hybrid scaffolds. (**a**) Guided differentiation of NSCs towards oligodendrocytes on GO-coated PCL nanofiber hybrid scaffolds. (i) Schematic illustration depicting the fabrication and application of GO-PCL nanofiber hybrid scaffolds. (ii) SEM images of NSCs on PCL and GO-PCL nanofiber scaffolds. Scale bars are 2 μm for upper row and 10 μm for lower row. (iii) Fluorescence images of NSCs on GO-PCL nanofiber scaffolds after 6 days of culture. Cells were stained for the early oligodendrocyte marker Olig2 and the mature oligodendrocyte marker myelin basic protein (MBP). (iv,v) Quantitative analysis of the expression of oligodendrocyte markers. Adapted with permission from Shah et al. [88]. Copyright (2014) John Wiley and Sons. (**b**) Promoted differentiation of embryonic neural progenitor cells into both neurons and glial cells on GO-based 3D porous scaffolds. (i) Digital photographs and (ii–vii) flexibility of GO-based 3D porous scaffolds. (viii) Neuronal differentiation and (ix) synapse formation of embryonic neural progenitor cells on GO-based 3D porous scaffolds. Reproduced with permission from [90]. Copyright The Royal Society of Chemistry, 2014.

On the other hand, recently, the natural and synthetic polymers have been extensively used to fabricate biological scaffolds for tissue engineering applications. Polymeric biomaterials have superior biocompatibility and biodegradability but their intrinsic poor thermal and mechanical properties are often quoted as disadvantages. Therefore, much research has been suggested to compensate the poor thermal and mechanical properties of polymeric biomaterials by functionalization of graphene possessing exceptional thermomechanical properties. Shin et al. demonstrated that the impregnation of GO can not only reinforced the poor mechanical properties of polymer nanofiber scaffolds but can also promote myoblast growth and differentiation [93]. Despite excellent biocompatibility of collagen-based scaffold, it suffers from poor mechanical and rapidly degrading properties of collagen. However, the poor mechanical properties, including tensile strength and elastic modulus, of scaffolds could be remarkably improved by the incorporation of GO. These improved mechanical properties of scaffolds could be rationalized by the fact that the oxygen-containing functional groups on GO surface can strongly interact with hydroxyl or amine groups of polymeric substrates, which allows interfacial bonding between GO and polymeric substrates [100,102,103,104,105]. Moreover, the cellular behaviors of myoblasts, including proliferation and myogenic differentiation, were significantly promoted on the GO-hybridized scaffolds [94,96]. As mentioned above, graphene nanomaterials have great capability in adsorption of serum proteins from culture media, which leads to accelerated myogenic differentiation [29,32]. Furthermore, the promoting effects of graphene nanomaterial-based hybrid scaffolds were confirmed in various types of cells. Shah et al. documented the guided differentiation of NSCs towards oligodendrocytes using GO-coated polycaprolactone (PCL) nanofiber scaffolds (Figure 10a); meanwhile Serrano et al. described the promoted differentiation of embryonic neural progenitor cells into both neurons and glial cells on GO-based scaffolds (Figure 10b) [88,90].

**Figure 11:**
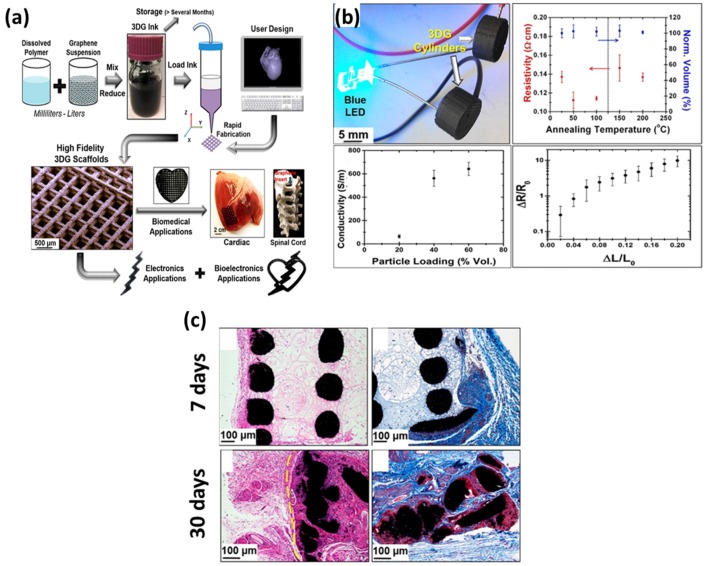
Feasibility of 3D printing for the fabrication of graphene-based 3D scaffolds. (**a**) Schematic diagram depicting the development of 3D printable graphene ink composed of PLGA and graphene flakes and the fabrication of 3D-printed graphene scaffolds. (**b**) Demonstration of the mechanical integrity and electrical conductivity in 3D-printed graphene scaffolds. (**c**) In vivo biocompatibility of 3D-printed graphene scaffolds. Reproduced with permission from [95]. Copyright American Chemical Society, 2015.

More recently, studies concerning the development of three-dimensional (3D) scaffolds using graphene nanomaterials have been increasingly reported [31,85,95,97,99,101]. It has been well known that the cellular behaviors, including migration, growth, morphology, differentiation and protein expression, are definitely different in 2D and 3D environments [106,107,108]. Thus, developing 3D scaffolds that mimic the in vivo microenvironment of the natural extracellular matrix is critical to biomedical applications. Jakus et al. suggested the feasibility of 3D printing for the fabrication of graphene-based 3D scaffolds (Figure 11) [95,99]. They developed 3D printable graphene ink composed of poly(lactic-co-glycolic acid, PLGA) and graphene flakes and fabricated 3D-printed graphene scaffolds. It was verified that the mechanical integrity and electrical conductivity of graphene were maintained in the 3D-printed graphene scaffolds and the viability and proliferation of human MSCs were significantly increased on the 3D-printed graphene scaffolds as compared to the PLGA scaffolds. In addition, the expression of neurogenic relevant genes, such as glial fibrillary acidic protein, neuron-specific class III β-tubulin (Tuj1), nestin and microtubule-associated protein 2, was upregulated in human MSCs on 3D-printed graphene scaffolds. Further in vivo studies using a female BALB/c mouse model validated that the 3D-printed graphene scaffolds did not induce a severe immune response or fibrous capsule formation, indicating that the 3D-printed graphene scaffolds were highly biocompatible. These findings expand the versatility and applicability of graphene nanomaterials for emerging biomedical applications. Collectively, the graphene nanomaterial-based scaffolds can provide a great value for the potentials of graphene nanomaterials in biomedical applications.

## 5. Conclusions

Splendid progress in nanoscience and nanotechnology has invigorated interest in application of nanomaterials to biomedical fields. Thanks to this interest, there has also been tremendous advances in research on graphene nanomaterials and their biocompatibility and biofunctionality having been gradually established. In this review, some of recent literature concerning the multifaceted biomedical applications of functional graphene nanomaterials was summarized and discussed. According to the recent studies, it is obvious that the functional graphene nanomaterials can be employed in a variety of ways to biomedical applications. In addition, the conventional approaches can be further developed by unique properties of graphene nanomaterials themselves, such as exceptional thermomechanical, excellent physicochemical, outstanding electrical and specific biological properties. In particular, many studies support the fact that graphene nanomaterial-coated substrates, -patterned arrays and hybrid scaffolds are typical approaches for biomedical applications of graphene nanomaterials, which allows us to more easily and intuitively understand the potential of graphene nanomaterials.

Although we have focused on three ways to apply graphene nanomaterials in biomedical applications, there are many other ways to employ them in biomedical fields and the potentials for application of graphene nanomaterials to biomedical fields will continue to evolve. Even if more research remains a significant challenge that should be addressed through comprehensive and systematic studies to fundamentally understand the functional graphene nanomaterials, we envision that the functional graphene nanomaterials will become promising novel candidates, which can open the way to handle unsolved problems in the current biomedical field.

## Figures and Tables

**Table 1:** Summary of studies on the various biomedical applications of graphene nanomaterial-coated substrates to date.

Applications	Target	Graphene Nanomaterial	Methods & Findings	Control	Ref.
Immunosensor	PSA-ACT	rGO on amine-SAM substrate	- Solution gated FET & anti-PSA - 100 fg/mL of detection limit	CEA	[60]
Immunosensor	*E. coli*	TRMGO on SiO_2_/Si	- Back gated FET & anti-*E. coli* - 10 cfu/mL of detection limit	Non-pathogenic *E. coli* & plant-pathogenic bacterium	[61]
Immunosensor	DNA	PNA-rGO on SiO_2_/Si	- Liquid-gated FET - Label-free detection - 100 fM of detection limit	Non-complementary DNA	[62]
Electrode array	Neural imaging	Four-layer graphene on Au or Pt	- Implantable on the brain surface in rodents - Ability of in vivo 3D imaging & optogenetic stimulation	Pt micro-ECoG device	[64]
Antibacterial system	*E. coli* & *S. aureus*	GONWs	- Achieving GONWs by EPD of Mg^2+^-GONSs - Toxic effects of GONWs on bacteria	RGNWs	[65]
GBR membrane	Rat calvarial defect	GO on Ti membrane	- Biocompatibility of GO at 10 μg/mL - Bone regeneration effects of GO-Ti	Ti membrane	[50]
Bone graft material	BMSCs & Rat calvarial defect	rGO-HA & rGO-BCP	- Osteogenetic effects of rGO - Bone regeneration ability of rGO	HA & BCP	[51,52]

Abbreviations: ACT, α1-antichymotrypsin; BCP, biphasic calcium phosphate; BMSC, bone marrow-derived mesenchymal stem cell; CEA, carcinoembryonic antigen; DNA, deoxyribonucleic acid; ECoG, electrocorticography; *E. coli*, *Escherichia coli*; EPD, electrophoretic deposition; FET, field-effect transistor; GBR, guided bone regeneration; GO, graphene oxide; GONS, graphene oxide nanosheet; GONW, graphene oxide nanowall; HA, hydroxyapatite; Non-pathogenic *E. coli*, *E. coli* DH5α; Plant-pathogenic bacterium, *Dickeya dadantii* 3937; PNA, peptide nucleic acid; PSA, prostate specific antigen; RGNW, reduced graphene nanowall; rGO, reduced graphene oxide; SAM, self-assembly monolayer; *S. aureus*, *Staphylococcus aureus*; 3D, three-dimensional; TRMGO, thermally reduced monolayer graphene oxide.
